# Climate Change Alters Seedling Emergence and Establishment in an Old-Field Ecosystem

**DOI:** 10.1371/journal.pone.0013476

**Published:** 2010-10-18

**Authors:** Aimée T. Classen, Richard J. Norby, Courtney E. Campany, Katherine E. Sides, Jake F. Weltzin

**Affiliations:** 1 Department of Ecology and Evolutionary Biology, University of Tennessee, Knoxville, Tennessee, United States of America; 2 Environmental Sciences Division, Oak Ridge National Laboratory, Oak Ridge, Tennessee, United States of America; 3 USA National Phenology Network, Tucson, Arizona, United States of America; Trinity College Dublin, Ireland

## Abstract

**Background:**

Ecological succession drives large-scale changes in ecosystem composition over time, but the mechanisms whereby climatic change might alter succession remain unresolved. Here, we asked if the effects of atmospheric and climatic change would alter tree seedling emergence and establishment in an old-field ecosystem, recognizing that small shifts in rates of seedling emergence and establishment of different species may have long-term repercussions on the transition of fields to forests in the future.

**Methodology/Principal Findings:**

We introduced seeds from three early successional tree species into constructed old-field plant communities that had been subjected for 4 years to altered temperature, precipitation, and atmospheric CO_2_ regimes in an experimental facility. Our experiment revealed that different combinations of atmospheric CO_2_ concentration, air temperature, and soil moisture altered seedling emergence and establishment. Treatments directly and indirectly affected soil moisture, which was the best predictor of seedling establishment, though treatment effects differed among species.

**Conclusions:**

The observed impacts, coupled with variations in the timing of seed arrival, are demonstrated as predictors of seedling emergence and establishment in ecosystems under global change.

## Introduction

Predicting the response of ecosystems — especially ecosystems in transition — to projected climatic change is a compelling but formidable challenge. Some models predict and research support that global change driven changes in plant community composition may be more important to regional terrestrial productivity and its feedback to the climate than will physiological responses of individual plant taxa [1], [2], [3]. However, few studies have experimentally examined the effects of multiple climatic drivers on ecosystems in transition [4], [5], [6]. Hence, it is important to measure the responses of transitional ecosystems under realistic simulations of climatic change and seek new approaches for generalizing from experimental observations.

Succession from abandoned agricultural and grassland ecosystems to forested ecosystems represents a key transition in ecosystem structure and function, and these transitions have the potential to generate large carbon sinks [7], [8]. Climatic change, such as warming and altered precipitation regimes, is causing shifts in species distributions [9], [10] and phenologies [11]. These changes can alter forest composition; for example, warming could increase the growth rate of established individuals or select for warm-adapted species. Although shifts in plant species distributions have been observed [12], [13], [14], we know little about the mechanisms behind these transitions [15], [16], [17], which limits our ability to predict future responses.

We investigated whether multiple climate change factors would alter the successional trajectory associated with woody plant establishment in an old-field ecosystem. We introduced seeds from three early successional tree species into constructed old-field plant communities that had been subjected for 4 years to altered temperature, precipitation, and atmospheric CO_2_ regimes in an experimental facility. The experiment was a randomized, complete block, split plot design where atmospheric CO_2_ concentrations (ambient or +300 ppm) and air temperature (ambient or +3°C) were applied at the plot level and precipitation (dry or wet) was applied at the split plot within each treatment. Atmospheric [CO_2_], air temperature, and precipitation were all main effects. Allowing the experiment to run for 4 years prior to the introduction of the tree seeds enabled the plant and soil communities to respond to the treatments [18], [19], [20], [21]. The tree seeds we used are common early successional species found invading old fields adjacent to our research site in the southeastern US: *Pinus taeda* (loblolly pine), *Liquidambar styraciflua* (sweetgum), and *Acer saccharinum* (silver maple).

Population demographics of woody plants establishing within intact successional plant communities are likely to be constrained by factors that alter germination and seedling establishment, and ultimately, recruitment of individuals into the population [22], [23], [24]. Light and water are the two most important abiotic determinants of seedling establishment and success [25]. The three environmental factors being manipulated in this experiment, atmospheric [CO_2_], air temperature, and precipitation, alter light availability and soil moisture, either directly or indirectly via changes in the plant community; hence, they might also influence the establishment of woody species in these old-field ecosystems [5]. Previous research in this experimental ecosystem documented that the plant community (changes in biomass and composition) to all three factors; however the change in biomass and in species composition was greatest in the precipitation treatments and precipitation explained most of the variation in plant community composition among treatments [20]. Thus, we predicted that soil moisture would exert the largest influence on seedling emergence and establishment and that fewer seedlings would emerge and establish in drier plots. Reduced soil moisture availability often acts as a barrier for seedling establishment in transitional ecosystems [e.g., [26], [27]. We also predicted that because seedling emergence and establishment happens at the soil surface where CO_2_ concentrations are relatively high and temperatures in established plant communities fluctuate less, warming and elevated CO_2_ concentrations would affect seedling emergence and establishment via their indirect impacts on soil moisture and the cover of the surrounding old-field plant community [27], [28]. Although temperature can have direct effects on seed germination and seedling carbon balance, we expect that direct effects of warming will be relatively smaller than indirect effects caused by warming-induced reductions in soil moisture [29]. Soil moisture at the microhabitat scale is a primary determinant of seedling establishment [30]. Hence, deleterious effects of warming should be more prevalent in drier plots than in wetter plots.

## Methods

### Site Description

The experimental facility was constructed at the Global Change Field Research Facility on the Oak Ridge National Environmental Research Park (35° 54′ 12″ N, 84° 20′ 22″ W). Site characteristics and experimental design have been previously described [31], [32] and are summarized briefly here. The experiment was conducted as a randomized, complete block, split-plot design. Four plots (4 m diameter) were laid out in each of three blocks in 2002. Open-top chambers were used to maintain the four treatment combinations of ambient or elevated (ambient + 300 ppm) atmospheric CO_2_ crossed with ambient or warmed (ambient + 3°C) air temperatures. Differential watering treatments were maintained on two split plots within each chamber by excluding rain with overhead shelters and irrigating with collected rainwater at 2 mm each week (dry) or 25 mm each week (wet). Each chamber contained a mix of seven old-field plant species including C_3_ and C_4_ grasses (*Andropogon virginicus* L., *Festuca pratense* L. syn *Festuca elatior* L., *Dactylis glomerata* L.), N-fixing herb and shrub (*Trifolium pratense* L., *Lespedeza cuneata* (Dum. Cours.) G. Don.), and forbs (*Plantago lanceolata* L., *Solidago canadensis*) [see [19], [20]. Environmental conditions, including air temperature and humidity, soil temperature, and soil moisture at multiple depths, were monitored routinely as previously described [29]. The integrated 0–15 cm vertical soil moisture probes represent the most relevant soil moisture profile for individual seedlings, and thus were used in this analysis. We visually estimated green foliar cover (%) for each of the seven old-field plant species within each subplot based on a modified Domin-Krajina cover scale [see 19,20]. To capture temporal dynamics of plant species, foliar cover data were recorded monthly for the 2007 growing season (March - November).

In the fall of 2006, loblolly pine and sweetgum seeds were collected from trees near the research site. In order to increase the likeliness of emergence in a large-scale experiment with a low sample size (n = 3), seeds were collected from half-sib populations (they share a mother) and preconditioned to mimic the essential process that occur in nature over winter. The species used in this study all occur naturally in the area surrounding the study and are common early successional species in Tennessee old fields. The experiment initially included *Quercus alba*, but these data were excluded due to a high rate of seed predation prior to excluding rodents. Pine seeds were scarified in sulfuric acid and stratified in moist peat or sand at 4°C for 90 days. Laboratory germination tests determined the number of seed planted in each location within the chambers to reduce the impact of unviable seeds. On 23 February, four loblolly pine seeds were planted adjacent to each other just beneath the soil surface in each of 10 locations per split plot, and on 2 March, 10 sweetgum seeds were planted adjacent to each other in each of 10 locations per split plot. Silver maple seeds, which mature in the spring, were collected from a single tree and planted in the chambers on 13 April, one seed in each of 10 locations per split plot. The planting locations were distributed on a 15×15 cm grid with alternate planting where groups of seeds (pine, sweetgum, silver maple) were planted in locations every 15 cm. It looked as though they were planted on a sheet of grid paper where every line is 15 cm long and seeds were planted at each intersection. Seeds were protected from rodent predation with small inverted cones made of 6 mm hardware cloth that was removed immediately following seedling emergence. Seedling surveys started on 30 March 2007 and concluded when all the plants senesced on 5 December 2007. For the emergence data, surveys were conducted two times a week until no new seedlings emerged.

As seeds began to germinate at the end of March, a census was taken twice weekly through July and weekly thereafter to determine emergence and mortality. To decrease interactions among closely emerged seedlings, seedlings were thinned to two per location on 23 April. Seedling emergence in each split plot was calculated as a percentage of seed initially planted, and includes seedlings that subsequently died or were thinned out. Seedling mortality was calculated as the percentage of emerged seedlings that died over the next year. Seedling establishment was considered to be the number of live seedlings the following spring as a percentage of the number of seeds planted, except that for pine and sweetgum the initial seed population was reduced by the number of seeds that emerged but were subsequently removed by thinning.

### Statistical analyses

The effects of species, [CO_2_], warming, and water treatments on emergence, mortality, and establishment were analyzed using a mixed-model, split-plot ANOVA (Proc MIXED, SAS Institute, Inc., Cary, NC). Species, [CO_2_], warming, and water and their interactions were fixed effects, and blocks and the interaction between blocks, [CO_2_], and warming were included as random effects (n = 3). Since species was a significant effect, separate analyses were done in the same framework for each species. We specified the Kenward-Roger method to estimate the denominator degrees of freedom for tests of the fixed effects.

To better describe the various environmental influences on emergence and establishment, we analyzed the emergence and establishment of each species independently in a multiple regression framework. Potential predictors included soil temperature (10 cm), soil moisture (0–15 cm), air temperature, daytime relative humidity (or vapor pressure deficit), measured CO_2_ concentration during the period when 80% emergence occurred, and old-field plant cover (sum of the percent cover of the seven species) near the midpoint of the emergence period. For each species, all possible regressions between the trait (emergence or establishment) or the natural logarithm of the trait of each species and the potential predictors (or their logarithms) were calculated across the 24 split-plots. Regressions with highly correlated (R>0.75) predictors (e.g., air temperature and vapor pressure deficit) were excluded. The best regression equation for each species-train combination was chosen based on Mallow's Cp statistic [33], [34].

## Results

### Seedling emergence and mortality

**Figure 1:**
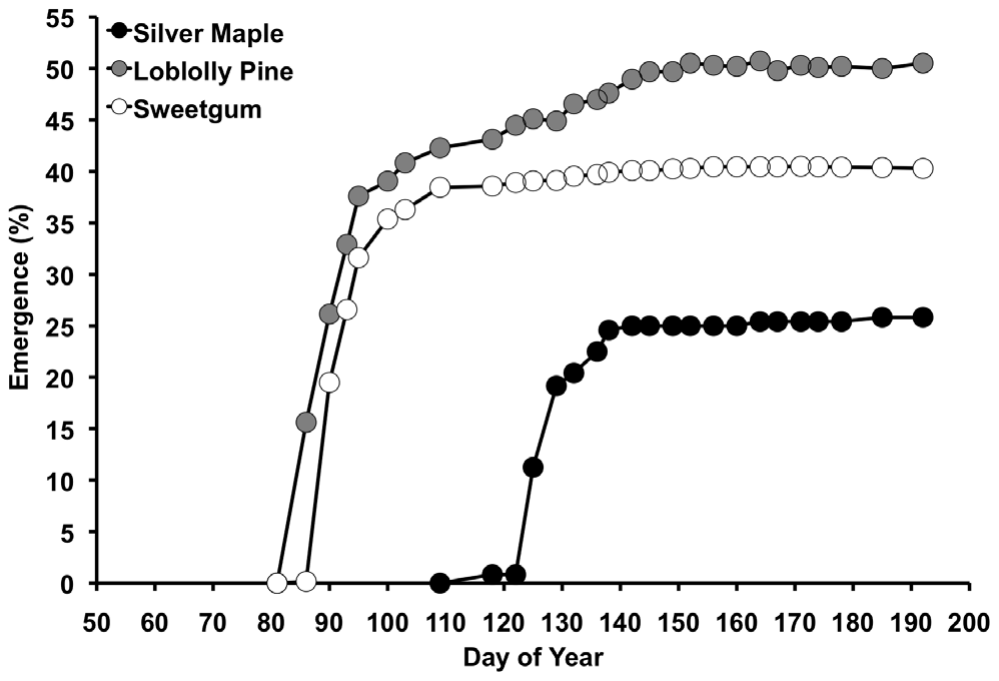
Seedling emergence. Silver maple seedlings, with a later, spring seed arrival, emerged one month after loblolly pine and sweetgum seedlings that have an earlier, fall seed arrival.

Tree seedlings from all three species emerged in the spring following their native pattern of seed dispersal and germination. Loblolly pine and sweetgum seeds, which overwintered, began to emerge in early spring. Of the seeds that were planted, 40–50% emerged (Fig. 1), and 80% of those that emerged did so by early April. Silver maple seeds, which do not overwinter, began to germinate and emerge shortly after they were planted; 80% emergence was achieved by early May, though total emergence was about half that of the other two species. There were no treatments effects on mortality, defined as the percentage of emerged seedlings that subsequently died.

### Microclimate and plant community response

**Figure 2:**
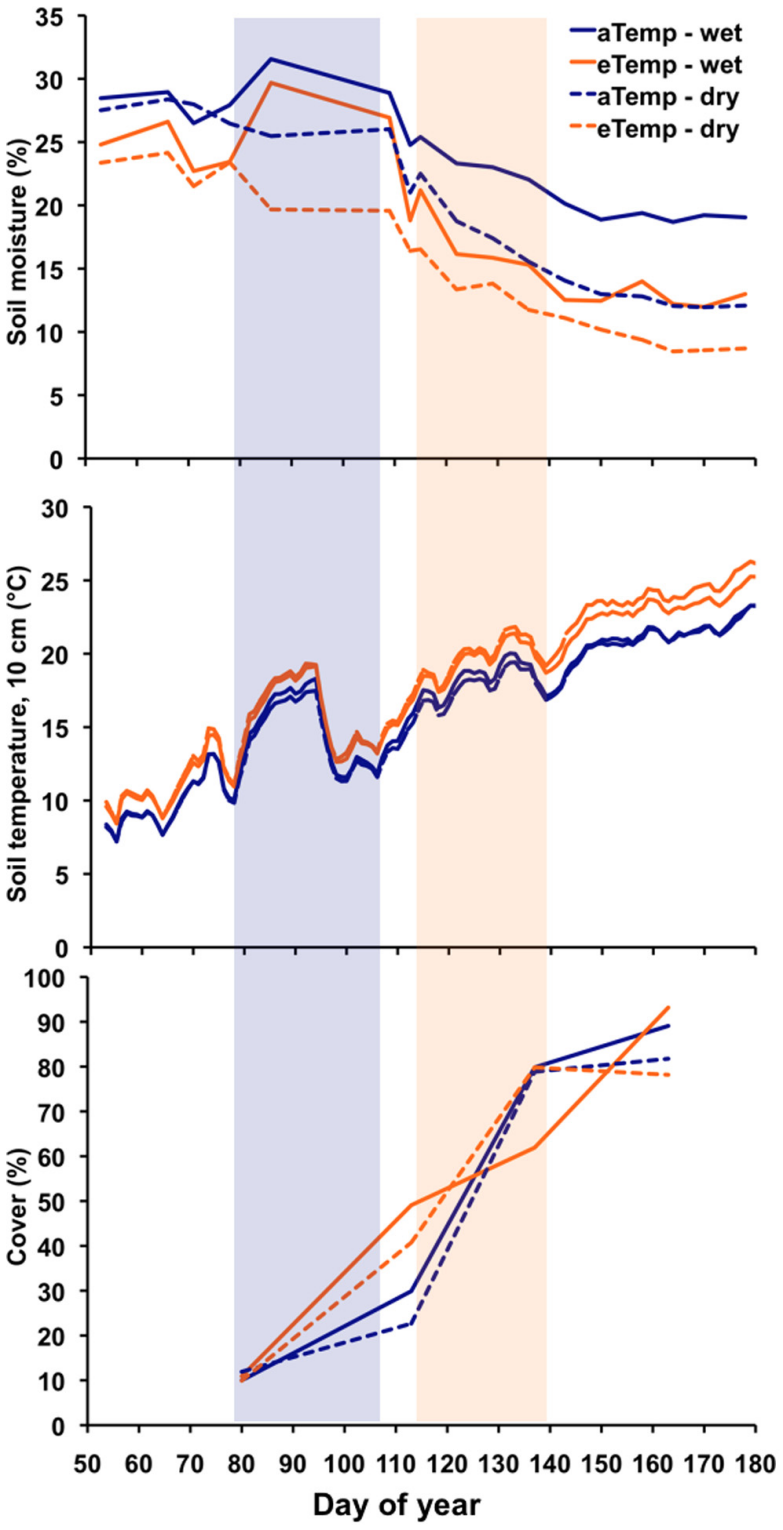
Experimental climate change impacts on ecosystem attributes. Soil moisture and temperature treatments became more pronounced and foliar cover of the old-field plant community increased as maple seedling emerged and established later in the spring. The first bar represents when pine and sweetgum seedlings emerged, while the second bar represents when maple seedlings emerged.

During the period when pine and sweetgum seedlings were emerging and establishing, soil moisture was relatively high, soil temperature was relatively low, and there was low foliar cover from the old-field plant community (Fig. 2). In contrast, one month later when maple seedlings were establishing, soil moisture was declining relative to earlier in the season, soil temperature was increasing, and the plant community cover was increasing.

### Treatment effects on emergence

**Figure 3:**
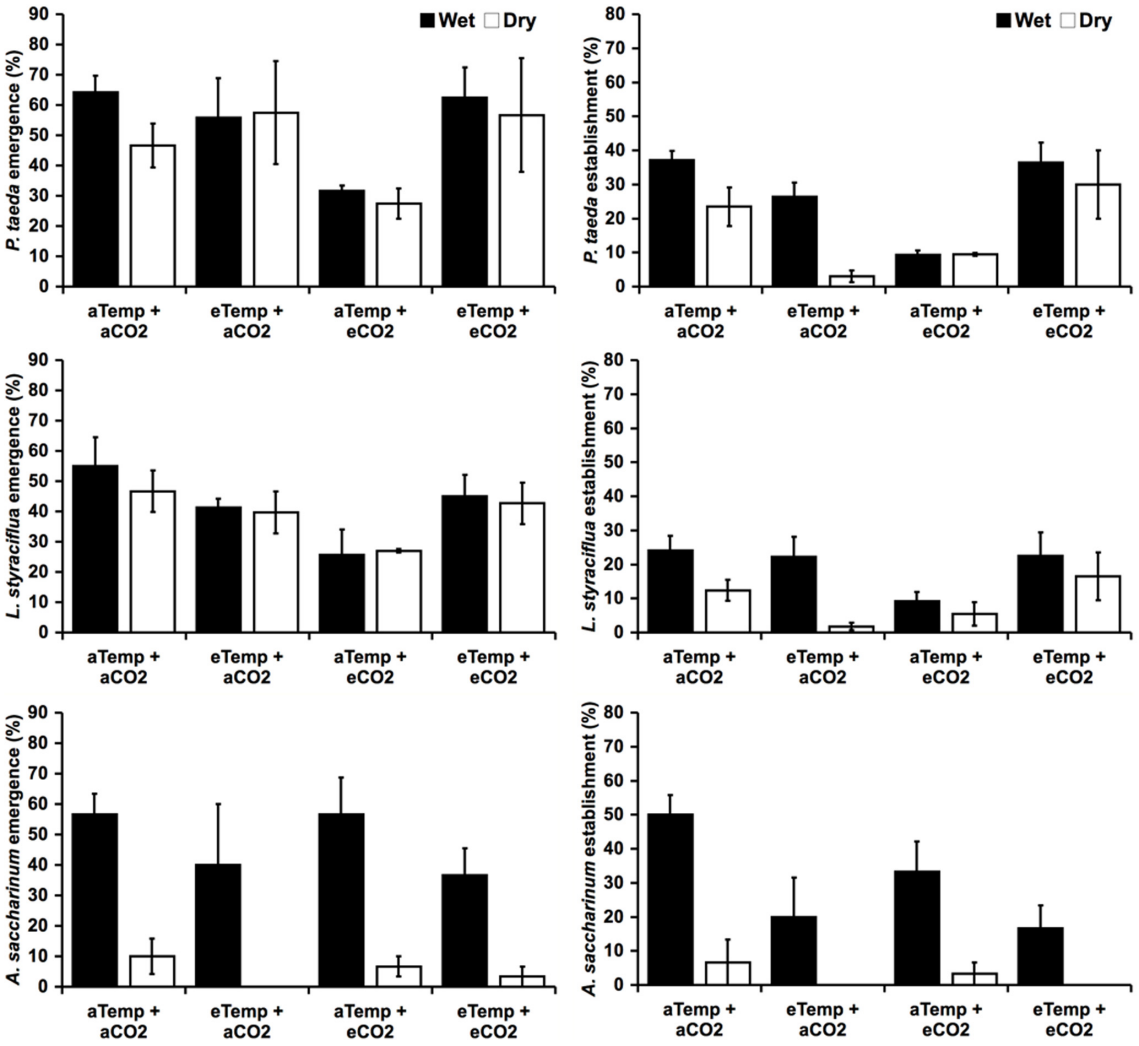
Species emergence and establishment. Emergence and establishment (%) of loblolly pine (*Pinus taeda*), sweetgum (*Liquidambar styraciflua*), and silver maple (*Acer saccharinum*) under the [CO_2_], temperature (temp), and water treatments. Data are means ± SE (n = 3). Abbreviations are: aTemp, ambient temperature; eTemp, elevated temperature; aCO_2_, ambient atmospheric [CO_2_]; and eCO_2_, elevated atmospheric [CO_2_].

**Figure 4:**
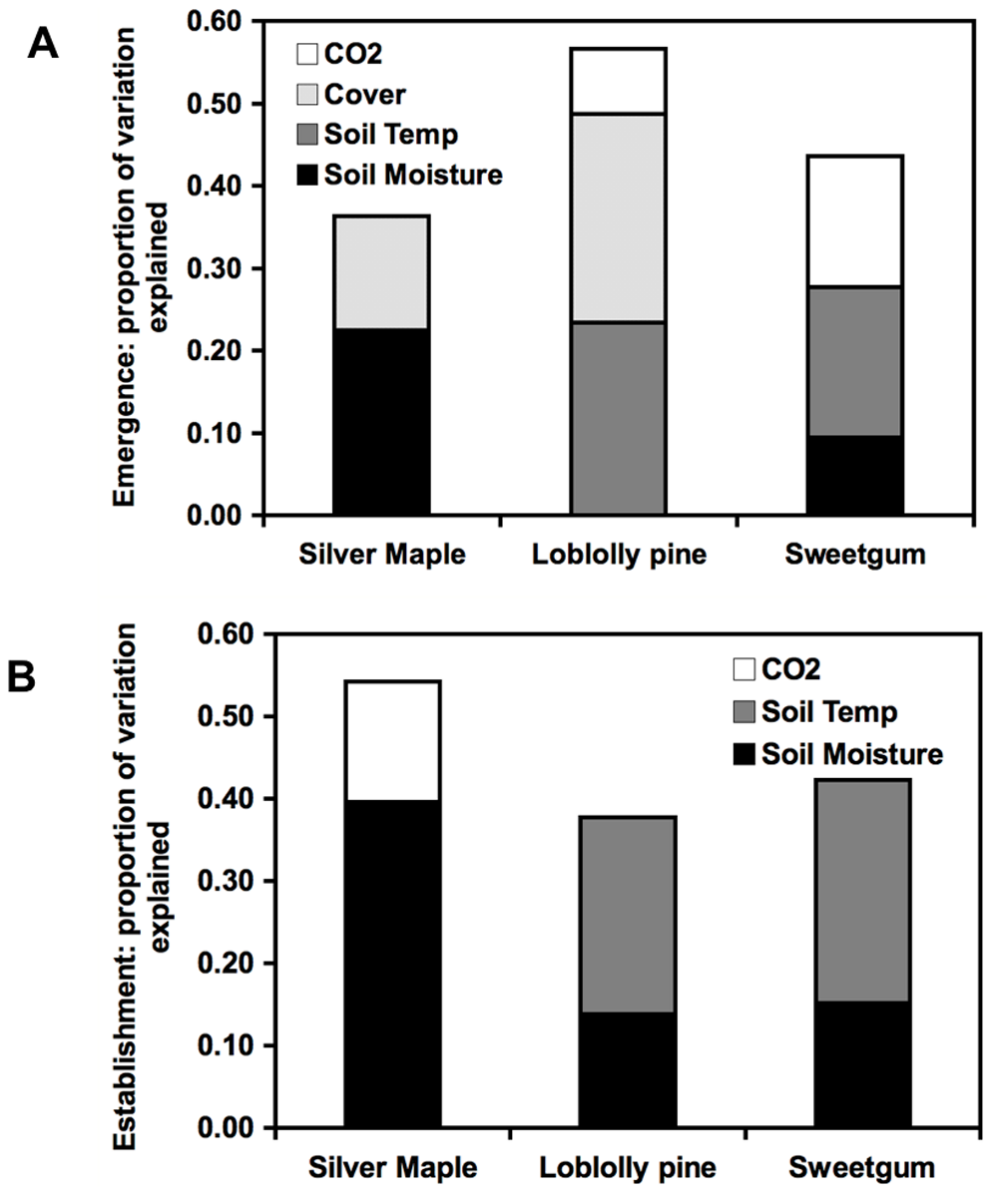
The proportion of variation that explains species emergence and establishment. Significant predictors of loblolly pine, sweetgum, and silver maple emergence and establishment.

Across species, emergence exhibited differential response to the watering and warming treatments (P<0.0001, P<0.008, respectively; Table 1). Differential watering had no significant effect on loblolly pine and sweetgum seedling emergence; in contrast, silver maple emergence was very low in dry treatments (P<0.001; Fig. 3; Table 2). Only five seedlings out of 120 planted silver maple seeds emerged in dry plots; soil moisture was the most significant predictor of silver maple seedling emergence, explaining 23% of the variation (P = 0.004; Fig. 4; Table 3).

**Table 1:** Results of an ANOVA examining the direct and interactive effects of changes in species, [CO_2_], air temperature, and soil moisture on seedling emergence, mortality, and establishment (n = 3).

	Emergence		Mortality		Establishment	
CO_2_	P = 0.15	F = 2.74	P = 0.30	F = 1.25	P = 0.20	F = 1.70
Temperature	P = 0.65	F = 0.24	P = 0.69	F = 0.17	P = 0.30	F = 1.11
CO_2_ × temperature	P = 0.08	F = 4.43	P = 0.14	F = 2.77	**P<0.0001**	F = 24.68
Water	**P<0.0001**	F = 24.71	P = 0.46	F = 0.56	**P<0.0001**	F = 53.56
CO_2_ × water	P = 0.66	F = 0.19	P = 0.22	F = 1.55	**P** = **0.01**	F = 6.88
Temperature × water	P = 0.30	F = 1.11	P = 0.76	F = 0.10	P = 0.73	F = 0.12
CO_2_ × temperature × water	P = 0.61	F = 0.26	P = 0.83	F = 0.05	P = 0.99	F = 0.00
Species	**P<0.0001**	F = 16.22	P = 0.09	F = 25.8	**P** = **0.02**	F = 4.26
CO_2_ × species	P = 0.39	F = 0.96	P = 0.31	F = 1.22	P = 0.65	F = 0.44
Temperature × species	**P<0.008**	F = 5.52	P = 0.78	F = 0.25	**P<0.003**	F = 7.08
CO_2_ × temperature × species	P = 0.21	F = 1.64	P = 0.09	F = 2.57	P = 0.02	F = 4.23
Water × species	**P<0.0001**	F = 13.37	P = 0.28	F = 1.32	**P** = **0.004**	F = 6.36
CO_2_ × water × species	P = 0.99	F = 0.01	P = 0.71	F = 0.35	P = 0.81	F = 0.21
Temperature × water × species	P = 0.83	F = 0.19	P = 0.12	F = 1.95	**P** = **0.04**	F = 3.58
CO_2_ × temperature × water × species	P = 0.66	F = 0.43	P = 0.46	F = 0.78	P = 0.73	F = 0.32

Bold numbers indicated statistical significance (P<0.05).

**Table 2:** Results of an ANOVA examining the direct and interactive effects of changes in [CO_2_], air temperature, and soil moisture on silver maple, sweetgum, and loblolly pine seedling emergence and establishment (n = 3).

	Loblolly pine		Sweetgum		Silver maple	
	Emergence	Establishment	Emergence	Establishment	Emergence	Establishment
**CO_2_**	P = 0.16	P = 0.12	**P<0.03**	P = 0.64	P = 0.91	P = 0.29
	F = 2.19	F = 0.74	F = 6.32	F = 0.23	F = 0.01	F = 1.36
**Temperature**	P = 0.06	P = 0.26	P = 0.41	P = 0.41	P = 0.13	**P = 0.03**
	F = 4.08	F = 1.39	F = 0.72	F = 0.74	F = 2.92	F = 8.03
**CO_2_ × temperature**	P = 0.08	**P<0.001**	**P = 0.005**	**P = 0.03**	P = 0.91	P = 0.45
	F = 3.45	F = 31.89	F = 10.92	F = 6.99	F = 0.01	F = 0.69
**Water**	P = 0.42	**P = 0.007**	P = 0.52	**P = 0.01**	**P<0.001**	**P<0.001**
	F = 0.70	F = 9.77	F = 0.43	F = 10.88	F = 49.08	F = 41.88
**CO_2_ × water**	P = 0.85	**P = 0.04**	P = 0.60	P = 0.11	P = 0.89	P = 0.36
	F = 0.04	F = 4.86	F = 0.29	F = 3.16	F = 0.02	F = 0.96
**Temperature × water**	P = 0.58	P = 0.26	P = 0.86	P = 0.41	P = 0.36	P = 0.06
	F = 0.32	F = 1.34	F = 0.03	F = 0.76	F = 0.92	F = 4.65
**CO_2_ × temperature × water**	P = 0.51	P = 0.82	P = 0.55	P = 0.63	P = 0.69	P = 0.35
	F = 0.45	F = 0.05	F = 0.38	F = 0.25	F = 0.17	F = 0.57

Bold numbers indicated statistical significance (P<0.05).

**Table 3:** Results of a multiple regression examining the effects of soil temperature, soil moisture, [CO_2_], and plant cover on seedling emergence and establishment.

Variable	Parameter	Partial r^2^	F	Probability
**Emergence**				
Loblolly Pine: P. taeda*				
Model		0.57	8.70	0.001
Soil temperature (°C)	4.23	0.23	9.82	0.005
Cover (%)	0.54	0.25	13.65	0.001
[CO_2_]	−0.48	0.08	3.64	0.071
Sweetgum: L. styraciflua*				
Model		0.44	5.16	0.008
Soil temperature (°C)	4.14	0.18	8.76	0.007
Soil moisture (VWC, 0–15 cm)	0.60	0.09	3.35	0.082
[CO_2_]	−0.63	0.16	7.15	0.015
Silver maple: *A. saccharinum*				
Model		0.36	5.97	0.009
Soil moisture (VWC, 0–15 cm)	3.56	0.23	10.62	0.004
Cover (%)	0.71	0.14	4.55	0.450
**Establishment**				
Loblolly Pine: P. taeda*				
Model		0.38	6.36	0.007
Soil temperature (°C)	9.56	0.24	8.06	0.010
Soil moisture (VWC, 0–15 cm)	2.20	0.14	10.99	0.003
Sweetgum: L. styraciflua*				
Model		0.42	7.68	0.003
Soil temperature (°C)	7.87	0.27	9.86	0.005
Soil moisture (VWC, 0–15 cm)	1.64	0.15	12.80	0.002
Silver maple: *A. saccharinum*				
Model		0.54	12.48	<0.001
[CO_2_]	−0.06	0.15	6.77	0.017
Soil moisture (VWC, 0–15 cm)	3.64	0.40	14.41	0.001

Treatment effects on emergence of the other species were subtle compared to maple. There was a significant temperature × species interaction (P<0.008; Table 1), where warming had little effect on loblolly pine and sweetgum emergence, but silver maple emergence was lower in warmed plots. There was no effect of warming, elevated CO_2,_ or water on pine emergence. Warming decreased sweetgum emergence in ambient CO_2_ treatments and increased emergence in elevated CO_2_ treatments (significant CO_2_ × warming interaction, P = 0.005; Fig. 3; Table 2). When examining the data using a multiple regression (Fig. 4; Table 3), 18% of the variation in sweetgum emergence and 23% of the variation in pine emergence was explained by soil temperature (P = 0.007 and P = 0.005, respectively). Total plant community cover explained another 25% of pine emergence (P = 0.001; Fig. 4; Table 3).

### Treatment effects on establishment

Across species, establishment exhibited differential response to water, temperature, and CO_2_; there were significant water × species (P = 0.004), temperature × species (P<0.003), temperature × water × species (P = 0.04), CO_2_ × temperature (P<0.0001), and CO_2_ × water interactions (P = 0.01; Table 1). Differential watering and warming treatments exerted the strongest influence on seedling establishment across species (Fig. 3; Table 2).

Maple establishment was 11-fold greater in wet relative to dry treatments (P<0.001); warming reduced establishment of maple (P = 0.03; Fig. 3; Table 2). Soil moisture explained 40% of the variation in maple establishment (P = 0.001; Fig. 3; Table 3), suggesting that the deleterious effects of warming are due to a reduction in soil moisture. Sweetgum establishment was higher in warm elevated CO_2_ treatments relative to elevated CO_2_ treatments with ambient temperatures (P = 0.03; Fig. 3; Table 2); and there establishment was significantly higher in wet relative to dry treatments (P = 0.01; Fig. 3; Table 2). Soil temperature explained 24% of the variation (P = 0.002) and soil moisture explained 14% of the variation in sweetgum establishment (P = 0.005; Fig. 4; Table 3). Pine establishment was higher in wet relative to dry treatments at ambient CO_2_, but there was no effect of water in the elevated CO_2_ treatments (P = 0.04; Fig. 3; Table 2); establishment was a 66% higher in wet relative to dry treatments (P = 0.007; Fig. 3; Table 2). Warming decreased pine establishment in ambient CO_2_ treatments, while it increased pine establishment under elevated CO_2_ (P<0.001; Fig. 3; Table 2). When examining establishment using a multiple regression, 24% of the variation in pine establishment was explained by soil temperature (P = 0.01), and soil moisture explained another 14% of the variation (P = 0.003; Fig. 3; Table 3).

## Discussion

Climatic change is clearly altering communities in old-field and forested ecosystems [35], [36]. For example, elevated atmospheric [CO_2_] can directly alter the quality of resources plants deliver to or take up from the soil [37], [38], while warming may increase soil nutrient availability [39], and decreases in soil moisture may alter plant biomass production [20]. Multiple climate change factors, such as changes in [CO_2_], temperature, and precipitation regimes, can interact in ways that make predicting their direct impact on ecosystems difficult [40]. Elevated atmospheric CO_2_ may increase plant production, but this response could be constrained by reductions in precipitation and soil moisture [20]. These interactions are further complicated by the indirect impacts of atmospheric and climatic change via shifts in plant community composition, which can result from changes in individual plant function (e.g., altered plant-competitive interactions) or from changes in individual plant distribution (e.g., species range shifts) [20], [21]. While a number of studies have measured how single species may respond to atmospheric and climatic changes, we know little about how ecosystems transitioning from fields to forest may respond. In this study, we find that climatic changes both directly and indirectly alter seedling emergence and establishment and that changes in soil moisture, either directly from our manipulation or due to other climatic change factors, often had the most influence on seedling emergence and establishment across species, though treatment effects differed among species.

### Interactive effects of soil moisture, temperature, and atmospheric [CO_2_] on seedling emergence and establishment

Silver maple, sweetgum, and loblolly pine are all early successional species that often colonize moist floodplains and bottomland areas, thus we would expect their emergence and establishment to be influenced by soil moisture availability. Soil moisture exerts strong control on a variety of community and ecosystem processes in this experiment [e.g., 18,19,20,21,41] and others [e.g., 25,42]; thus its importance in predicting seedling emergence and establishment is not surprising, and these results support our prediction. Interestingly, the emergence and establishment of one species, silver maple, which has a different timing of seed dispersal, was dramatically reduced by soil moisture availability relative to the other two species. In fact, treatment effects on the emergence of the other species were subtle compared to maple.

It is unlikely that CO_2_ concentration had any direct effect on germination and seedling emergence—processes unlikely to benefit from direct effects of CO_2_ on photosynthesis [43]; and seedlings emerge at the soil surface, where concentrations of CO_2_ are already high due to soil respiration [32]. The effects of elevated atmospheric [CO_2_] on seedling emergence and establishment are likely indirect, mediating soil temperature or moisture via past or current effects on the plant or soil community. Warming reduces soil moisture at this site and its impact is greater in dry plots; however, elevated atmospheric CO_2_ concentrations reduce this effect by leading to an increase in soil moisture [29]. Other work at this site indicates that the indirect impacts of CO_2_ and temperature can alter the both the plant and soil community [18], [19], [20], [21], [31], [41] as well as the interaction between grasses and symbiotic endophytes [44]. Further, total plant foliar cover during the periods of seedling emergence explained 25% and 14% of the variation in pine and maple emergence, respectively, suggesting that changes in plant community structure influences emergence [25].

Water played a large role in governing seedling establishment across species; establishment of all three species was lowest in dry treatments. Overall, our results supported our predictions that (1) warming would affect seedling establishment primarily through its effects on soil moisture because the indirect effects of warming on soil moisture will be greater than the direct effects of warming on seedling establishment, and that (2) deleterious effects of warming would be more prevalent under dry than wet conditions. Our prediction was supported—temperature and soil moisture were significant predictors of establishment; however, the effect of warming was not always deleterious to establishment. For example, when elevated [CO_2_] was combined with warming, seedling emergence was higher. It is possible that elevated [CO_2_] ameliorated the negative impacts of warming on soil moisture [29]. Silver maple was the only species whose establishment was significantly reduced by warming.

Plant life history traits and their response to environmental variation are increasingly recognized as important predictors of how communities might respond to climatic change [45], [46]. We propose that the differential response of seedling emergence and establishment to the experimental treatments were controlled in part by different seed phenology and that this trait might be important in predicting a species response to climatic change. Pine and sweetgum seeds mature in the fall and germinate in early spring after over-wintering, whereas maple seeds mature in late spring and germinate soon after they fall. During the time sweetgum and pine were germinating and establishing soil moisture was relatively high across all treatments. In contrast, during the critical time period for maple seedling emergence and establishment, air and soil temperatures were relatively high, and soil moisture was relatively low; thus, differences in soil moisture and warming had a much greater impact on emergence and establishment of maple than on pine or sweetgum. Thus, seed phenology linked to environmental conditions can impact the probability of emergence and establishment, and changes in environment are likely to either ameliorate or exacerbate seedling emergence and establishment without concomitant shifts in phenology.

Successful seedling establishment is controlled by a series of plant traits such as production, dispersal, and germination and environmental filters including seed herbivory, microbial symbiosis, community history, and early seedling growth and survival [e.g., [47], [48], [49], [50], [51]. These traits and filters are likely to be affected by different abiotic influences, and their relationships can be expected to change under a changing climate. For example, light and water are the two most important abiotic factors driving seedling establishment and success and both will be influenced, directly or indirectly, by atmospheric and climate-driven changes in temperature and precipitation regimes. Our results suggest that differences in seedling emergence and establishment may depend on seed traits and their interaction with dynamic soil environments and plant community composition. Other early successional woody species that have seeds with a spring-maturing early phenology (e.g., *Acer rubrum*, *Ulmus* spp., *Salix* spp., *Populus* spp.) may be more susceptible to climate change than seeds with a fall-maturing late phenology. Previous research demonstrates that early germination by species can increase establishment in some ecosystems by reducing pathogen pressure [52], [53], thus understanding how these advantages interact with climatic change would be important. Nonetheless, our data suggest that understanding changes in soil moisture, whether directly related to changes in precipitation or indirectly though changes in temperature or plant cover, will be critical when forecasting what species will be able to establish in the future.

The encroachment of trees and other woody plants into grasslands and abandoned fields, which is recognized as an important landscape-scale process and a key uncertainty in carbon budgets [8], [54], could be altered if new environmental conditions influence the successful emergence and establishment of woody plant seedlings. While previous experiments have focused on how established grassland and forested ecosystems respond to global changes, this experiment focused on an ecosystem in transition to demonstrate that atmospheric and climatic change can alter the dynamics of ecosystem succession, and potentially, carbon sequestration. We suggest that seed traits, including phenology, should be considered as an important ecological reality in future experimental designs and possibly during the development of earth system and dynamic vegetation models that have recently incorporated other characteristics such as growth and mortality [6]. Moreover, interactions among phenology and multiple atmospheric or climatic driving variables that influence woody plant emergence may ultimately affect successional trajectories resulting in forests with structure and function quite different from those of present forests [43], [54]. Clearly, more work needs to be done to better understand and predict how seed traits of different species, such as phenology, interact with climate drivers to alter seedling emergence, establishment, and ecosystem succession.
